# Predicting Urosepsis in Ureteral Calculi: External Validation of Hu’s Nomogram and Identification of Novel Risk Factors

**DOI:** 10.3390/diagnostics15091104

**Published:** 2025-04-26

**Authors:** Yuka Sugizaki, Takanobu Utsumi, Naoki Ishitsuka, Takahide Noro, Yuta Suzuki, Shota Iijima, Takatoshi Somoto, Ryo Oka, Takumi Endo, Naoto Kamiya, Hiroyoshi Suzuki

**Affiliations:** Department of Urology, Toho University Sakura Medical Center, Sakura 285-8741, Japan; yuuka.kizuki@med.toho-u.ac.jp (Y.S.); naoki.ishitsuka@med.toho-u.ac.jp (N.I.); takahide.noro@med.toho-u.ac.jp (T.N.); yuta.suzuki@med.toho-u.ac.jp (Y.S.); shouta.iijima@med.toho-u.ac.jp (S.I.); takatoshi.soumoto@med.toho-u.ac.jp (T.S.); ryou.oka@med.toho-u.ac.jp (R.O.); takumi.endou@med.toho-u.ac.jp (T.E.); naoto.kamiya@med.toho-u.ac.jp (N.K.); hiroyoshi.suzuki@med.toho-u.ac.jp (H.S.)

**Keywords:** external validation, nomogram, ureteral calculi, urosepsis, risk prediction

## Abstract

**Background/Objectives**: Acute obstructive pyelonephritis caused by ureteral calculi is a severe urological emergency that can rapidly progress to life-threatening complications, including urosepsis. Early risk stratification is critical for timely intervention and improved patient outcomes. Although Hu’s nomogram has been proposed as a predictive tool for urosepsis, its external validation remains limited. This study aims to validate Hu’s nomogram in an independent cohort and identify novel clinical and imaging predictors of urosepsis. **Methods**: This retrospective cohort study included 341 patients diagnosed with ureteral calculi who underwent surgical intervention at a single institution between January 2019 and October 2023. Clinical, laboratory, and imaging data were collected. Univariate and multivariate logistic regression analyses were performed to identify independent predictors of urosepsis. The predictive accuracy of Hu’s nomogram was evaluated using receiver operating characteristic curve analysis. **Results**: Among 341 patients, 66 (19.4%) developed urosepsis. Multivariate analysis identified female gender, corticosteroid use, lower platelet count, elevated C-reactive protein levels, positive urine white blood cell count, lower computed tomography attenuation values of calculi, and higher computed tomography attenuation values of hydronephrosis as independent predictors of urosepsis. Hu’s nomogram demonstrated a strong predictive performance (area under the curve: 0.761; 95% CI: 0.701–0.821), reaffirming its clinical utility for risk stratification. **Conclusions**: This study provides an external validation of Hu’s nomogram and identifies novel risk factors for urosepsis prediction, including corticosteroid use and imaging-based parameters. Incorporating these findings into clinical practice may enhance early risk stratification, facilitate timely interventions, and ultimately improve patient outcomes.

## 1. Introduction

The prevalence of urinary stones has shown a continuous upward trend over recent decades [1,2]. However, over the past ten years, the incidence of urinary stones in Japan has remained relatively stable. The annual incidence of upper urinary tract stones is estimated to be 138 per 100,000 individuals (192 per 100,000 in men and 87 per 100,000 in women), closely mirroring prevalence rates reported in Europe and the United States [1,3]. Despite advancements in diagnostic imaging such as computed tomography (CT) and the increasing use of minimally invasive surgical techniques, the early detection and effective management of obstructive pyelonephritis remain significant clinical challenges [1,2,4,5].

Acute obstructive pyelonephritis (AOPN) due to ureteral calculi is a severe and potentially life-threatening urological condition, characterized by a high infectious burden resulting from urinary tract obstruction [4,5]. Without prompt diagnosis and timely intervention, this condition can rapidly progress to serious complications, including urosepsis, disseminated intravascular coagulation (DIC), and septic shock [6,7]. Mortality rates associated with severe sepsis and septic shock remain substantial, ranging from 22% to 76% worldwide, reflecting disparities in healthcare resources and management strategies [4,5,6,7].

The optimal management of AOPN requires the urgent decompression of the obstructed urinary tract, typically performed via ureteral stent placement or percutaneous nephrostomy, alongside targeted antimicrobial therapy [2,4,5]. However, standardized criteria for determining the optimal timing and necessity of these interventions remain undefined and vary across institutions. In this context, predictive models, particularly nomograms designed to estimate the risk of urosepsis in patients with ureteral calculi, have emerged as valuable clinical tools to support risk assessment and decision-making.

Recent studies have emphasized the importance of early risk stratification in improving patient outcomes [2,8]. Hu et al. identified key predictive factors for urosepsis in patients hospitalized with ureteral calculi, including gender, CT attenuation values of hydronephrosis, urine white blood cell (WBC) count, urine nitrite positivity, and the presence of a functional solitary kidney. Their predictive nomogram highlights the potential of personalized risk assessment to facilitate early intervention and reduce the incidence of urosepsis [8]. However, its applicability to external patient populations remains unvalidated, necessitating further research.

Considering the significant clinical and economic burden of AOPN-associated urosepsis, the development of robust, evidence-based management protocols is imperative. Accurate diagnosis and prompt intervention are paramount in mitigating both the healthcare impact and economic burden associated with this condition. This study aims to identify the clinical predictors associated with urosepsis secondary to AOPN in patients diagnosed with ureteral calculi at our institution while concurrently conducting an external validation of Hu’s nomogram to evaluate its predictive accuracy. Furthermore, we compare the prognostic utility of the identified clinical predictors with that of the nomogram, assessing its relative effectiveness in risk stratification.

## 2. Materials and Methods

### 2.1. Study Design and Population

This retrospective cohort study was conducted at Toho University Sakura Medical Center, a high-volume regional referral center specializing in the management of urinary tract stones and infections. The study cohort comprised all patients diagnosed with ureteral calculi who underwent transurethral lithotripsy (TUL) between January 2019 and October 2023. A total of 341 Japanese patients were included, consisting of 66 patients diagnosed with urosepsis and 275 without urosepsis.

To assess the risk of AOPN-related urosepsis, we applied Hu’s predictive nomogram, originally developed to estimate sepsis risk based on a rapid escalation in the sequential (sepsis-related) organ failure assessment (SOFA) score, with a threshold of ≥2 points [8]. In this study, systemic inflammatory response syndrome (SIRS) criteria were employed to define sepsis, wherein SIRS in the presence of a suspected infection was classified as sepsis. While SIRS demonstrates high sensitivity but relatively low specificity, it remains widely used in clinical practice due to its simplicity and ease of application [9].

SIRS was diagnosed when patients met at least two of the following four criteria: (1) body temperature abnormality (greater than 38 °C or less than 36 °C), (2) tachycardia (greater than 90 beats per minute), (3) respiratory abnormality (respiratory rate greater than 20 breaths per minute or PaCO_2_ less than 32 Torr), (4) WBC count abnormality (WBC count greater than 12,000 cells/mm^3^, less than 4000 cells/mm^3^, or more than 10% immature forms or bands) [9,10]. By employing this approach, we aimed to maintain consistency in sepsis risk assessment and validate the applicability of Hu’s nomogram within our institutional setting.

### 2.2. Data Collection

This study aimed to identify key risk factors contributing to urosepsis in patients with ureteral calculi. Comprehensive clinical data were meticulously extracted from electronic medical records, encompassing demographic variables (gender, age, and body mass index [BMI]), performance status, and medical history, including urolithiasis, diabetes mellitus, and hyperparathyroidism. Additionally, information on medication use, including anticoagulant/antiplatelet agents and corticosteroid therapy, was recorded. The clinical characteristics of ureteral calculi, such as maximum stone size, laterality, and the presence of ipsilateral renal stones, were also documented.

Laboratory parameters included WBC count, hemoglobin (Hb), platelet count, C-reactive protein (CRP), estimated glomerular filtration rate (eGFR), serum albumin, serum uric acid, corrected serum calcium, hemoglobin A1c (HbA1c), urine pH, urine WBC count, and urine nitrite levels. Urine WBC count was assessed using a urinary reagent strip method (semi-quantitative dipstick test) and categorized as follows: 0 = negative, +1 = 10–20 WBC/HPF, +2 = 20–50 WBC/HPF, and +3 = >50 WBC/HPF. Kidney-related factors, such as the severity of hydronephrosis and the presence of a functional solitary kidney, were incorporated into the dataset.

For imaging-based parameters, the mean CT attenuation values of both ureteral calculi and hydronephrosis were measured using our institution’s Picture Archiving and Communication System. Hydronephrosis severity was classified according to the congenital hydronephrosis grading system as follows [11]: Grade I: dilation limited to the renal pelvis; Grade II: dilation of the renal pelvis and a few calyces; Grade III: significant dilation of the renal pelvis and all calyces; and Grade IV: severe dilation of the renal pelvis and calyces, with thinning of the renal parenchyma.

The inclusion criteria for this study were aligned with those established by Hu et al. [8], including the following: (1) a confirmed diagnosis of ureteral calculi based on imaging modalities such as CT; (2) the availability of complete laboratory and imaging data; (3) the diagnosis of sepsis based on SIRS criteria. All patients included in the study had complete datasets, ensuring data integrity without missing values.

### 2.3. Statistical Analysis

Complete baseline data from 341 Japanese patients diagnosed with ureteral calculi were analyzed to identify clinical predictors associated with the progression to urosepsis. First, univariate analyses were conducted to assess associations with urosepsis development across the entire cohort. Parametric and non-parametric variables were examined using t-tests and Mann–Whitney U-tests, respectively, while categorical variables were analyzed using χ^2^-tests or Fisher’s exact test, as appropriate. Second, variables that demonstrated significant associations in the univariate analyses were incorporated into multivariate logistic regression models to identify independent predictors of urosepsis progression. Multivariate logistic regression analysis was conducted to identify independent predictors of urosepsis, incorporating variables that demonstrated statistical significance in univariate analyses.

Third, the predictive performance of Hu’s nomogram was assessed using receiver operating characteristic (ROC) curve analysis, with the area under the curve (AUC) value used to determine its discriminative ability [12]. Lastly, AUC values from Hu’s nomogram were compared with AUC values from individual predictors identified in univariate analyses to determine their relative predictive efficacy. A *p* value < 0.05 was considered statistically significant. All statistical analyses were conducted using JMP Academic Suite (SAS Institute Inc., Cary, NC, USA) (https://www.jmp.com/en/software (accessed on 24 April 2025)) and EZR software (https://www.jichi.ac.jp/saitama-sct/SaitamaHP.files/statmedEN.html (accessed on 24 April 2025), version 1.68) [13].

### 2.4. Ethical Considerations

The study protocol was approved by the Ethics Committee of Toho University Sakura Medical Center (Approval No. S24084_S23020). Given the retrospective nature of this study, informed consent was waived. The study protocol was disclosed on the institutional website, allowing participants the opportunity to opt out. All procedures adhered to the ethical principles outlined in the Declaration of Helsinki.

## 3. Results

### 3.1. Patient Characteristics

This study included 341 patients diagnosed with ureteral calculi, among whom 66 (19.4%) developed urosepsis while 275 (78.7%) did not. The baseline characteristics of the study population are summarized in Table 1.

The proportion of female patients was significantly higher in the urosepsis group compared to those without urosepsis (59.1% vs. 32.4%, *p* < 0.001). The median age of the entire cohort was 62.0 years (IQR: 21.0 years), with a significantly higher median age observed in the urosepsis group (72.0 years vs. 60.0 years, *p* = 0.001).

Patients with an Eastern Cooperative Oncology Group Performance Status (ECOG-PS) of ≥2 had a significantly greater risk of developing urosepsis (15.2% vs. 2.2%, *p* < 0.001). Additionally, patients receiving corticosteroid therapy exhibited a markedly higher likelihood of developing urosepsis (7.6% vs. 1.8%, *p* = 0.027).

### 3.2. Laboratory and Imaging Findings

Laboratory analysis revealed that patients who developed urosepsis exhibited significantly elevated WBC levels (14,990/mm^3^ vs. 7080/mm^3^, *p* < 0.001), reduced hemoglobin concentrations (12.9 g/dL vs. 14.0 g/dL, *p* < 0.001), and lower platelet counts (16.6 × 10^4^/mm^3^ vs. 24.0 × 10^4^/mm^3^, *p* < 0.001). Additionally, they demonstrated markedly elevated CRP levels (11.5 mg/dL vs. 0.3 mg/dL, *p* < 0.001), significantly lower eGFR (43.4 mL/min/1.73 m^2^ vs. 61.7 mL/min/1.73 m^2^, *p* < 0.001), and reduced serum albumin levels (3.6 g/dL vs. 4.1 g/dL, *p* < 0.001). Urinalysis findings indicated that positive urine WBC was significantly more prevalent in the urosepsis group (83.3% vs. 39.3%, *p* < 0.001). Similarly, urine nitrite positivity was strongly associated with urosepsis development (27.3% vs. 3.3%, *p* < 0.001).

Imaging analysis demonstrated that patients with urosepsis had significantly lower mean CT attenuation values of calculi (695.0 HU vs. 893.5 HU, *p* < 0.001) and higher mean CT attenuation values of hydronephrosis (11.5 HU vs. 7.4 HU, *p* = 0.001). However, no significant differences were observed in hydronephrosis severity (Grade III/IV) or the presence of a functional solitary kidney between the two groups.

### 3.3. Clinical Predictors of Urosepsis

Univariate analysis identified several factors that were significantly associated with urosepsis, including female gender (*p* < 0.001), advanced age (*p* = 0.001), ECOG-PS ≥2 (*p* < 0.001), corticosteroid use (*p* = 0.027), elevated WBC levels (*p* < 0.001), decreased hemoglobin levels (*p* < 0.001), reduced platelet counts (*p* < 0.001), increased CRP levels (*p* < 0.001), lower eGFR (*p* < 0.001), decreased serum albumin levels (*p* < 0.001), positive urine WBC (*p* < 0.001), positive urine nitrite (*p* < 0.001), lower CT attenuation values of calculi (*p* < 0.001), and higher CT attenuation values of hydronephrosis (*p* = 0.001) (Table 2).

As WBC count is a defining criterion of SIRS, it was excluded from our multivariate analysis. In the multivariate logistic regression analysis, the following factors emerged as independent predictors of urosepsis: female gender (OR: 2.506; 95% CI: 1.036–6.061; *p* = 0.041), corticosteroid use (OR: 8.130; 95% CI: 1.548–41.667; *p* = 0.013), lower platelet count (OR: 1.059; 95% CI: 1.004–1.119; *p* = 0.034), higher CRP levels (OR: 1.328; 95% CI: 1.221–1.445; *p* < 0.001), positive urine WBC (OR: 4.695; 95% CI: 1.712–12.820; *p* = 0.003), lower CT attenuation values of calculi (OR: 1.002; 95% CI: 1.000–1.003; *p* = 0.018), and higher CT attenuation values of hydronephrosis (OR: 1.154; 95% CI: 1.078–1.235; *p* < 0.001) (Table 2).

### 3.4. Predictive Performance of Hu’s Nomogram and Each Significant Predictor

To assess the external validity of Hu’s nomogram, it was applied to our institutional dataset. Receiver operating characteristic (ROC) curve analysis demonstrated an AUC of 0.761 (95% CI: 0.701–0.821), indicating good predictive performance.

**Figure 1:**
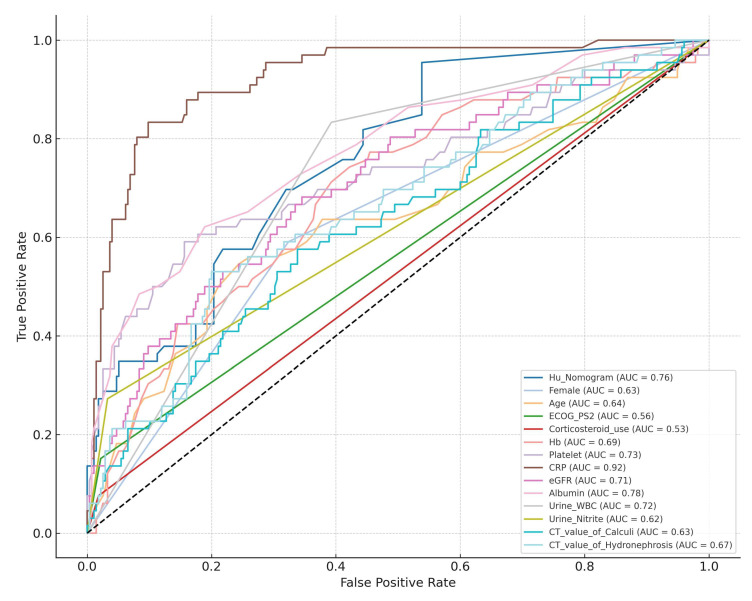
Comparative analysis of AUC values between Hu’s Nomogram and individual predictive variables.

The AUC values of each significant predictor identified through univariate analyses are presented in Table 3. Compared individually, Hu’s nomogram significantly outperformed predictors such as the following: female gender, age, ECOG-PS ≥2, corticosteroid use, urine nitrite positivity, CT attenuation values of calculi, and CT attenuation values of hydronephrosis (Table 3 and Figure 1).

However, the nomogram did not demonstrate significantly higher predictive accuracy compared to Hb, platelet count, eGFR, albumin, or urine WBC levels. Notably, CRP exhibited superior predictive performance compared to the nomogram (Table 3 and Figure 1).

## 4. Discussion

AOPN secondary to ureteral calculi is a life-threatening urological emergency that necessitates early risk stratification and timely intervention to prevent severe complications such as urosepsis, septic shock, and DIC [4,5,7,14]. In emergency settings, the rapid assessment of AOPN risk and the potential for sepsis progression is imperative, necessitating urgent consultation with a urologist to determine the indication for ureteral stent placement or nephrostomy. Our study identified independent predictors of urosepsis and externally validated Hu’s nomogram, a predictive model designed to assess urosepsis risk in patients with ureteral calculi [8]. Our findings demonstrated that Hu’s nomogram exhibited strong predictive performance in an external cohort (AUC: 0.761), reaffirming its clinical utility for risk stratification. By utilizing the simpler and more clinically accessible SIRS criteria rather than the more complex SOFA score to assess disease severity [15], this nomogram is expected to facilitate timely and appropriate drainage interventions, mitigating the risk of severe urinary tract infections and improving patient outcomes. This nomogram enables a comprehensive, multidimensional assessment of the patient’s condition, extending beyond the limitations of SIRS criteria alone.

Several studies have sought to establish reliable risk factors for obstructive pyelonephritis and urosepsis [8,16,17,18,19,20]. Consistent with prior research, univariate analysis in our study identified advanced age, female gender, poor ECOG-PS, elevated WBC count, lower platelet count, higher CRP levels, reduced eGFR, lower serum albumin, positive urine WBCs, positive urine nitrite, and higher CT attenuation values of hydronephrosis as significant predictors of urosepsis. Additionally, our study highlights corticosteroid use, lower hemoglobin levels, and lower CT attenuation values of calculi as novel predictive factors, suggesting that immunosuppression, patient frailty, and stone composition may contribute to urosepsis risk.

Elderly patients, individuals with limited physical activity, and those who are immunosuppressed—such as patients receiving corticosteroids or immunosuppressive therapy—are particularly susceptible to infection due to compromised host defense mechanisms [17]. Advanced age, female gender, and impaired performance status have been identified as significant risk factors necessitating emergency drainage in patients with upper urinary tract calculi [21]. Given their heightened susceptibility due to anatomical and hormonal influences, prior studies have reported that the risk of infection in females is approximately 1.8 times higher than in males [21].

Elevated WBC count, decreased platelet count, and increased CRP levels are well-established biomarkers of systemic inflammation and sepsis, commonly observed in AOPN-associated urosepsis. These parameters play a critical role in early risk stratification and help guide treatment decisions in emergency settings. However, additional laboratory markers should be considered when evaluating urosepsis risk comprehensively [22,23,24]. Although low Hb levels are not traditionally recognized as independent predictors of sepsis in obstructive pyelonephritis, evidence from related contexts suggests that anemia may exacerbate infection severity [25]. In patients undergoing surgical stone removal, preoperative anemia has been linked to increased susceptibility to postoperative SIRS. A 2022 study on percutaneous nephrolithotomy (PCNL) outcomes further reinforced this association, reporting that low Hb levels were significantly correlated with post-PCNL SIRS, aligning with the previous literature [25]. While Hb itself may not directly indicate urosepsis, it may reflect a broader patient profile of chronic illness and reduced physiological reserve, thereby increasing susceptibility to severe infection.

Regarding hydronephrosis, CT attenuation values have been shown to correlate with the risk of AOPN, with higher values indicating dense, infected fluid within the renal collecting system. This increases the likelihood of positive urine cultures and the development of pyelonephritis. Erdogan et al. reported that at a Hounsfield unit (HU) cutoff of 8.46, the sensitivity and specificity for diagnosing pyelonephritis were 68.4% and 92.6%, respectively, with a significantly higher urine culture positivity rate in the pyelonephritis group compared to those with hydronephrosis alone [26]. Similarly, Yuruk et al. identified a cutoff of 9.21 HUs, which could distinguish AOPN with a sensitivity of 65.96% and a specificity of 87.93%, further underscoring the utility of CT imaging in differentiating infectious from noninfectious hydronephrosis [27]. Furthermore, lower CT attenuation values of calculi suggest the presence of infected or porous stones, underscoring the importance of assessing both calculi and hydronephrosis when predicting urosepsis risk. This highlights the critical role of integrating imaging parameters into clinical decision-making, as they provide objective, quantitative data that may aid in early risk assessment and individualized treatment planning.

Urine nitrite, a key component of Hu’s nomogram, is a metabolic byproduct produced by bacteria with nitrate reductase activity, such as *Escherichia coli*. Previous studies have demonstrated that the presence of nitrites in urine serves as a reliable indicator of bacterial infection, frequently correlating with urinary tract infections [8,28]. The external validation of Hu’s nomogram in our study demonstrated good predictive accuracy, reinforcing its clinical utility as a real-world risk stratification tool.

Notably, higher CRP levels outperformed the nomogram, underscoring the importance of a multivariable approach in risk assessment (Figure 1). Among the predictors not included in the nomogram, lower Hb, reduced platelet count, decreased eGFR, and lower albumin levels emerged as good variables of urosepsis, as evidenced by their comparable AUC values to the nomogram (Table 3). This suggests that these parameters may enhance risk prediction models by capturing additional dimensions of patient frailty and systemic inflammation. A major strength of our study is its rigorous external validation of an established predictive model in a cohort of Japanese patients, providing critical insights into its applicability across different populations. Additionally, by incorporating both laboratory and imaging-based predictors, our study offers a comprehensive assessment of urosepsis risk factors.

This study identified critical predictors of urosepsis in patients diagnosed with ureteral calculi, categorizing them into two clinically relevant groups: patient vulnerability factors and markers of active infection and inflammation (Table 4). The patient vulnerability factors encompass advanced age, female gender, corticosteroid use, low Hb levels, reduced eGFR, and decreased serum albumin concentrations [29]. These vulnerability-related parameters collectively represent the patient’s physiological fragility or chronic health conditions, thereby increasing their susceptibility to severe infection and poor clinical outcomes. Advanced age and female gender, for instance, reflect an inherently higher susceptibility to urinary tract infections and diminished immunological defenses. Corticosteroid use may signify an impaired immune system response, elevating infection risk. Additionally, anemia, reflected by low Hb levels, can suggest underlying chronic diseases or malnutrition, potentially exacerbating infection severity. Interestingly, while bilateral ureteral obstruction and the presence of a functional solitary kidney were not significantly associated with urosepsis in this cohort, decreased eGFR emerged as an important vulnerability indicator, likely representing underlying chronic kidney impairment.

Conversely, the inflammatory and infectious markers identified include elevated WBC counts, thrombocytopenia, significantly elevated CRP levels, positive urine WBC counts, urine nitrite positivity, lower CT attenuation values of calculi, and higher CT attenuation values of hydronephrosis. These markers reflect acute systemic inflammation or ongoing bacterial infections, vital for identifying severe infectious processes early in clinical practice. Among these, elevated CRP levels exhibited particularly strong predictive capabilities, emphasizing their role as a critical biomarker of severe systemic inflammation in urological infections. To the best of our knowledge, this study is the first to introduce such a clinically meaningful framework distinguishing between patient vulnerability and inflammatory status (Table 4). Integrating this dual categorization into clinical practice can facilitate early recognition and targeted intervention, ultimately improving outcomes by minimizing progression to severe complications in patients with ureteral calculi.

However, our study has several limitations. First, its retrospective design introduces potential selection bias. Second, we did not include procalcitonin, presepsin, neutrophil count, neutrophil-to-lymphocyte ratio, or other emerging biomarkers, which could further enhance sepsis risk stratification [14,18,20]. Future studies should explore the integration of these biomarkers into predictive models. Third, although corticosteroid use was included as a proxy for immunosuppression and found to be an independent risk factor in our analysis, other immunosuppressive conditions such as malignancies, autoimmune disorders, or organ transplantation were not systematically evaluated. This limitation should be addressed in future studies to better understand the role of immunosuppression in urosepsis development. Finally, while our study demonstrated the good predictive performance of Hu’s nomogram, larger prospective multicenter studies are warranted to further refine and validate its clinical utility across diverse populations. By addressing these limitations and incorporating a broader spectrum of risk factors, future research should consider expanding upon our findings by exploring additional biomarkers, incorporating multicenter data, and evaluating the utility of modern machine learning models such as artificial neural networks and random forest algorithms. These approaches may enhance predictive performance by capturing complex, non-linear relationships among clinical variables.

## 5. Conclusions

This study provides a preliminary external validation of Hu’s nomogram and identifies key clinical and imaging predictors of urosepsis, categorizing patient vulnerability and markers of active infection and inflammation in individuals with ureteral calculi. Incorporating these findings into clinical practice may enhance early risk stratification, enable timely and targeted interventions, and ultimately reduce the incidence of severe complications, thereby improving patient outcomes.

## Figures and Tables

**Table 1:** Baseline characteristics of study cohort.

**Variables**	**Total (*N* = 341)**
Gender (male/female), *n* (%)	213 (62.5)/128 (37.5)
Age (years), median (IQR)	62.0 (21.0)
BMI (kg/m^2^), median (IQR)	24.1 (5.0)
ECOG-PS ≥2, *n* (%)	16 (4.7)
Medical history: Urolithiasis, *n* (%) Diabetes mellitus, *n* (%) Hyperparathyroidism, *n* (%)	176 (51.6) 64 (18.8) 8 (2.4)
Use of medications: Anticoagulant/antiplatelet agents, *n* (%) Corticosteroids, *n* (%)	26 (7.6) 12 (2.9)
Ureteral calculi: Max stone size (mm), median (IQR) Laterality (right/left/bilateral) Ipsilateral renal stones	8.5 (4.9) 183 (53.6)/138 (40.5)/20(5.9) 179 (52.4)
Blood test: WBCs (/mm^3^), median (IQR) Hb (g/dL), median (IQR) Platelets (×10^4^/mm^3^), median (IQR) CRP (mg/dL), median (IQR) eGFR (mL/min/1.73 m^2^), median (IQR) Albumin (g/dL), median (IQR) Uric acid (mg/dL), median (IQR) Correct Ca (mg/dL), median (IQR) HbA1c (%), median (IQR)	7670.0 (5075.0) 13.7 (2.5) 22.6 (9.5) 0.6 (5.1) 56.8 (27.6) 4.1 (0.6) 5.8 (2.0) 9.5 (0.6) 5.8 (0.8)
Urinalysis: Urine pH Urine WBCs (0/1+/2+/3+), *n* (%) Urine nitrite (positive), *n* (%)	6.5 (1.5) 178 (52.2)/65 (19.9)/45 (13.2)/53 (14.7) 27 (7.9)
Degree of hydronephrosis: (None/Grade I/Grade II/Grade III/Grade IV), *n* (%)	11 (3.2)/134 (39.3)/132 (38.7)/55 (16.1)/9 (2.7)
Functional solitary kidney, *n* (%)	3 (0.9)
Mean CT attenuation value (HU): Calculi, median (IQR) Hydronephrosis, median (IQR)	842.0 (490.0) 7.8 (7.0)
Sepsis, *n* (%)	66 (19.4)

BMI: body mass index; Ca: calcium; CRP: C-reactive protein; CT: computed tomography; eGFR: estimated glomerular filtration rate; ECOG-PS: Eastern Cooperative Oncology Group Performance Status; Hb: hemoglobin; HU: Hounsfield units; IQR: interquartile range; Max: maximum; WBCs: white blood cells.

**Table 2:** Uni- and multivariate analyses identifying independent predictors of urosepsis.

Variables	Sepsis (*n* = 66)	No Sepsis (*n* = 275)	*p* Value	Odds Ratio (95% CI)	*p* Value
Gender (female), *n* (%)	39 (59.1)	89 (32.4)	<0.001	2.506 (1.036–6.061)	0.041
Age (years), median (IQR)	72.0 (25.3)	60.0 (20.0)	0.001	-	0.712
BMI (kg/m^2^), median (IQR)	23.9 (5.6)	24.4 (5.0)	0.173		
ECOG-PS ≥2, *n* (%)	10 (15.2)	6 (2.2)	<0.001	-	0.467
Medical history: Urolithiasis, *n* (%) Diabetes mellitus, *n* (%) Hyperparathyroidism, *n* (%)	32 (48.5) 12 (18.2) 1 (1.5)	144 (52.4) 52 (18.9) 7 (2.6)	0.571 0.892 0.521		
Use of medications: Anticoagulant/antiplatelet agents, *n* (%) Corticosteroids, *n* (%)	6 (9.1) 5 (7.6)	20 (7.3) 5 (1.8)	0.617 0.027	8.130 (1.548–41.667)	0.013
Ureteral calculi: Max stone size (mm), median (IQR) Bilateral ureteral calculi Ipsilateral renal stones, *n* (%)	10.2 (10.7) 1 (1.5) 31 (47.0)	10.6 (9.2) 19 (6.9) 148 (53.8)	0.322 0.072 0.317		
Blood test: WBCs (/mm^3^), median (IQR) Hb (g/dL), median (IQR) Platelets (×10^4^/mm^3^), median (IQR) CRP (mg/dL), median (IQR) eGFR (mL/min/1.73 m^2^), median (IQR) Albumin (g/dL), median (IQR) Uric acid (mg/dL), median (IQR) Correct Ca (mg/dL), median (IQR) HbA1c (%), median (IQR)	14,990 (6890) 12.9 (2.3) 16.6 (12.9) 11.5 (10.5) 43.4 (31.6) 3.6 (1.1) 5.4 (2.9) 9.5 (0.7) 5.9 (1.0)	7080 (3540) 14.0 (2.3) 24.0 (8.2) 0.3 (1.6) 61.7 (28.1) 4.1 (0.6) 5.9 (1.9) 9.5 (0.5) 5.8 (0.7)	<0.001 <0.001 <0.001 <0.001 <0.001 <0.001 0.305 0.607 0.822	- - 1.059 (1.004–1.119) 1.328 (1.221–1.445) - -	- 0.940 0.034 <0.001 0.395 0.404
Urinalysis: Urine pH Urine WBCs (positive), *n* (%) Urine nitrite (positive), *n* (%)	6.5 (1.5) 55 (83.3) 18 (27.3)	6.5 (1.5) 108 (39.3) 9 (3.3)	0.621 <0.001 <0.001	4.695 (1.712–12.820) -	0.003 0.154
Degree of hydronephrosis: (Grade III/Grade IV), *n* (%)	8 (12.1)	56 (20.4)	0.124		
Functional solitary kidney, *n* (%)	2 (3.0)	1 (0.4)	0.097		
Mean CT attenuation value (HU): Calculi, median (IQR) Hydronephrosis, median (IQR)	695.0 (499.0) 11.5 (8.5)	893.5 (476.0) 7.4 (6.3)	<0.001 <0.001	1.002 (1.000–1.003) 1.154 (1.078–1.235)	0.018 <0.001

BMI: body mass index; Ca: calcium; CRP: C-reactive protein; CT: computed tomography; eGFR: estimated glomerular filtration rate; ECOG-PS: Eastern Cooperative Oncology Group Performance Status; Hb: hemoglobin; HU: Hounsfield units; IQR: interquartile range; Max: maximum; WBCs: white blood cells.

**Table 3:** Area under the curve metrics for individual predictors and Hu’s nomogram.

Predictors	AUC (95% CI)	*p* Value (Compared with Nomogram)
Hu’s nomogram	0.761 (0.701–0.821)	-
Gender (female)	0.634 (0.568–0.700)	<0.001
Age	0.638 (0.555–0.720)	0.009
ECOG-PS ≥ 2	0.565 (0.520–0.609)	<0.001
Corticosteroid use	0.529 (0.496–0.562)	<0.001
Hb	0.690 (0.618–0.763)	0.077
Platelets	0.729 (0.651–0.808)	0.511
CRP	0.924 (0.889–0.959)	<0.001
eGFR	0.708 (0.636–0.781)	0.256
Albumin	0.775 (0.708–0.842)	0.718
Urine WBCs	0.720 (0.667–0.774)	0.214
Urine nitrite	0.620 (0.565–0.675)	<0.001
Mean CT attenuation value of calculi	0.629 (0.552–0.705)	<0.001
Mean CT attenuation value of hydronephrosis	0.668 (0.594–0.742)	0.014

AUC: area under the curve; CI: confidence interval; CRP: C-reactive protein; CT: computed tomography; eGFR: estimated glomerular filtration rate; ECOG-PS: Eastern Cooperative Oncology Group Performance Status; Hb: hemoglobin; WBCs: white blood cells.

**Table 4:** Classification of key predictors of urosepsis according to clinical relevance.

Patient Vulnerability	Active infection and Inflammation
Age Female gender Corticosteroid use Low Hb levels Reduced eGFR Decreased serum albumin concentration	Elevated WBC counts Thrombocytopenia Elevated CRP levels Positive urine WBC counts Urine nitrite positivity Lower CT attenuation values of calculi Higher CT attenuation values of hydronephrosis

CRP: C-reactive protein; CT: computed tomography; eGFR: estimated glomerular filtration rate; Hb: hemoglobin; WBC: white blood cell.

## Data Availability

The original contributions presented in the study are included in the article, further inquiries can be directed to the corresponding author.

## Abbreviations

The following abbreviations are used in this manuscript:

APON	acute obstructive pyelonephritis
AUC	area under the curve
BMI	body mass index
CT	computed tomography
CRP	C-reactive protein
DIC	disseminated intravascular coagulation
eGFR	estimated glomerular filtration rate
EOCG-PS	Eastern Cooperative Oncology Group Performance Status
PCNL	percutaneous nephrolithotomy
SIRS	systematic inflammatory response syndrome
SOFA	sequential (sepsis-related) organ failure assessment
TUL	transurethral lithotripsy
WBC	white blood cell
